# (2,2′-Dimethyl-4,4′-bi-1,3-thia­zole-κ^2^ *N*,*N*′)bis(thio­cyanato-κ*S*)mercury(II)

**DOI:** 10.1107/S1600536809006904

**Published:** 2009-03-06

**Authors:** Nasser Safari, Vahid Amani, Anita Abedi, Behrouz Notash, Seik Weng Ng

**Affiliations:** a Department of Chemistry, General Campus, Shahid Beheshti University, Tehran, Iran; b Department of Chemistry, University of Malaya, 50603 Kuala Lumpur, Malaysia

## Abstract

The Hg^II^ atom in the title compound, [Hg(SCN)_2_(C_8_H_8_N_2_S_2_)], is chelated by the bidentate heterocycle through the N atoms and is coordinated by the S atoms of two thiocyanate anions, resulting in a considerably distorted tetra­hedral coordination geometry.

## Related literature

There are several examples of mercuric thio­cyanate–α,α′-dimine type of adducts which exist as four-coordinate, tetra­hedral mol­ecules. For the 4,4′,5,5′-tetra­methyl-2,2′-biimidazole adduct, see: Mahjoub *et al.* (2003 ▶); Morsali (2006 ▶). For the 2,2′-diamino-4,4′-bithia­zole adduct, see: Morsali *et al.* (2003 ▶). For the 2,2′-biquinoline adduct, see: Morsali *et al.* (2004 ▶); Ramazani *et al.* (2004 ▶). For the 2,2′-diphenyl-4,4′-bithia­zole adduct, see: Mahjoub & Morsali (2003 ▶).
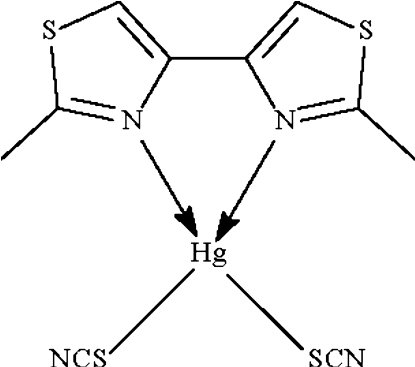


## Experimental

### 

#### Crystal data

[Hg(NCS)_2_(C_8_H_8_N_2_S_2_)]
*M* *_r_* = 513.03
Monoclinic, 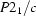

*a* = 17.3764 (3) Å
*b* = 12.0534 (2) Å
*c* = 7.0601 (1) Å
β = 100.676 (1)°
*V* = 1453.10 (4) Å^3^
*Z* = 4
Mo *K*α radiation
μ = 11.16 mm^−1^
*T* = 118 K
0.22 × 0.06 × 0.04 mm

#### Data collection

Bruker SMART APEX diffractometer
Absorption correction: multi-scan (*SADABS*; Sheldrick, 1996 ▶) *T*_min_ = 0.274, *T*_max_ = 0.640
10030 measured reflections
3330 independent reflections
2982 reflections with *I* > 2σ(*I*)
*R*_int_ = 0.030

#### Refinement

*R*[*F* ^2^ > 2σ(*F* ^2^)] = 0.022
*wR*(*F* ^2^) = 0.054
*S* = 1.04
3330 reflections
174 parameters
H-atom parameters constrained
Δρ_max_ = 1.17 e Å^−3^
Δρ_min_ = −1.32 e Å^−3^

### 

Data collection: *APEX2* (Bruker, 2008 ▶); cell refinement: *APEX2* (Bruker, 2008 ▶); data reduction: *SAINT*; program(s) used to solve structure: *SHELXS97* (Sheldrick, 2008 ▶); program(s) used to refine structure: *SHELXL97* (Sheldrick, 2008 ▶); molecular graphics: *X-SEED* (Barbour, 2001 ▶); software used to prepare material for publication: *publCIF* (Westrip, 2009 ▶).

## Supplementary Material

Crystal structure: contains datablocks global, I. DOI: 10.1107/S1600536809006904/xu2488sup1.cif

Structure factors: contains datablocks I. DOI: 10.1107/S1600536809006904/xu2488Isup2.hkl

Additional supplementary materials:  crystallographic information; 3D view; checkCIF report

## Figures and Tables

**Table:** 

Hg1—S3	2.413 (1)
Hg1—S4	2.421 (1)
Hg1—N1	2.430 (3)
Hg1—N2	2.476 (3)

**Table:** 

S3—Hg1—S4	149.25 (4)
S3—Hg1—N1	95.66 (8)
S3—Hg1—N2	105.84 (8)
S4—Hg1—N1	113.49 (8)
S4—Hg1—N2	94.04 (8)
N1—Hg1—N2	69.1 (1)

**Fig. 1.**
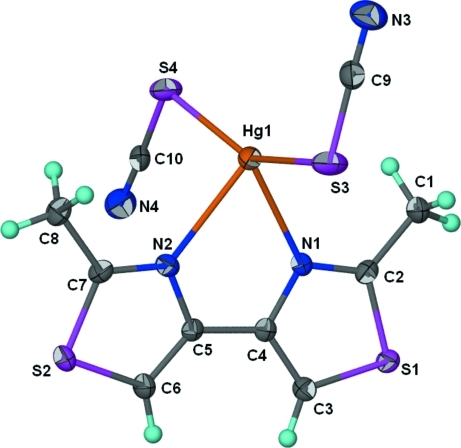
Thermal ellipsoid plot (Barbour, 2001) of Hg(SCN)2(C10H8N2S2); ellipsoids are drawn at the 70% probability level and H atoms of arbitrary radius.

## supplementary crystallographic information

### Experimental

A solution of 2,2'-dimethyl-4,4'-bithiazole (0.13 g, 0.66 mmol) in methanol (10 ml) was added to a solution of mercuric thiocyanate (0.21 g, 0.66 mmol) in methanol (5 ml). Crystals were obtained by diffusing the methanol solution into DMSO for a week (yield: 80%; m.p. 456 K).

### Refinement

Carbon-bound H-atoms were placed in calculated positions (C—H 0.95–0.98 Å) and were included in the refinement in the riding model approximation, with U_iso_(H) set to 1.2–1.5U_eq_(C).

The crystal diffracted strongly owing to the extremely heavy metal atom; however, its presence introduced severe absorption problems that could not be corrected analytically as the crystal did not have regular faces. The final difference Fourier map had a large peak/hole in the vicinity of the mercury atom.

### Crystal data

**Table:**

[Hg(NCS)_2_(C_8_H_8_N_2_S_2_)]	*F*(000) = 960
*M**_r_* = 513.03	*D*_x_ = 2.345 Mg m^−^^3^
Monoclinic, *P*2_1_/*c*	Mo *K*α radiation, λ = 0.71073 Å
Hall symbol: -P 2ybc	Cell parameters from 4390 reflections
*a* = 17.3764 (3) Å	θ = 2.4–28.3°
*b* = 12.0534 (2) Å	µ = 11.16 mm^−^^1^
*c* = 7.0601 (1) Å	*T* = 118 K
β = 100.676 (1)°	Block, colorless
*V* = 1453.10 (4) Å^3^	0.22 × 0.06 × 0.04 mm
*Z* = 4	

### Data collection

**Table:**

Bruker SMART APEX diffractometer	3330 independent reflections
Radiation source: fine-focus sealed tube	2982 reflections with *I* > 2σ(*I*)
graphite	*R*_int_ = 0.030
ω scans	θ_max_ = 27.5°, θ_min_ = 1.2°
Absorption correction: multi-scan (*SADABS*; Sheldrick, 1996)	*h* = −22→22
*T*_min_ = 0.274, *T*_max_ = 0.640	*k* = −15→15
10030 measured reflections	*l* = −8→9

### Refinement

**Table:**

Refinement on *F*^2^	Primary atom site location: structure-invariant direct methods
Least-squares matrix: full	Secondary atom site location: difference Fourier map
*R*[*F*^2^ > 2σ(*F*^2^)] = 0.022	Hydrogen site location: inferred from neighbouring sites
*wR*(*F*^2^) = 0.054	H-atom parameters constrained
*S* = 1.04	*w* = 1/[σ^2^(*F*_o_^2^) + (0.0213*P*)^2^ + 2.1611*P*] where *P* = (*F*_o_^2^ + 2*F*_c_^2^)/3
3330 reflections	(Δ/σ)_max_ = 0.001
174 parameters	Δρ_max_ = 1.17 e Å^−^^3^
0 restraints	Δρ_min_ = −1.31 e Å^−^^3^

### Fractional atomic coordinates and isotropic or equivalent isotropic displacement parameters (Å^2^)

**Table:**

	*x*	*y*	*z*	*U*_iso_*/*U*_eq_	
Hg1	0.272849 (8)	0.578858 (12)	0.74895 (2)	0.01665 (6)	
S1	0.37984 (5)	0.20867 (8)	0.65772 (15)	0.0156 (2)	
S2	0.03250 (6)	0.34937 (8)	0.73374 (16)	0.0192 (2)	
S3	0.34553 (7)	0.58014 (9)	1.07522 (18)	0.0287 (3)	
S4	0.20772 (7)	0.68035 (9)	0.46884 (17)	0.0241 (2)	
N1	0.30803 (18)	0.3905 (3)	0.6768 (5)	0.0132 (7)	
N2	0.16170 (18)	0.4504 (3)	0.7495 (5)	0.0147 (7)	
N3	0.4178 (2)	0.7908 (3)	1.1095 (6)	0.0317 (9)	
N4	0.1421 (2)	0.4986 (3)	0.2409 (6)	0.0270 (8)	
C1	0.4453 (2)	0.4223 (3)	0.6435 (7)	0.0221 (9)	
H1A	0.4274	0.4900	0.5719	0.033*	
H1B	0.4807	0.3815	0.5755	0.033*	
H1C	0.4730	0.4421	0.7729	0.033*	
C2	0.3765 (2)	0.3514 (3)	0.6592 (6)	0.0149 (8)	
C3	0.2837 (2)	0.2028 (3)	0.6837 (6)	0.0148 (8)	
H3	0.2549	0.1364	0.6912	0.018*	
C4	0.2545 (2)	0.3082 (3)	0.6921 (6)	0.0128 (8)	
C5	0.1758 (2)	0.3395 (3)	0.7151 (5)	0.0124 (7)	
C6	0.1123 (2)	0.2727 (3)	0.7042 (6)	0.0163 (8)	
H6	0.1121	0.1949	0.6834	0.020*	
C7	0.0890 (2)	0.4686 (3)	0.7608 (6)	0.0158 (8)	
C8	0.0558 (2)	0.5793 (3)	0.7950 (7)	0.0207 (9)	
H8A	0.0831	0.6090	0.9186	0.031*	
H8B	−0.0001	0.5716	0.7980	0.031*	
H8C	0.0626	0.6301	0.6910	0.031*	
C9	0.3879 (2)	0.7054 (3)	1.0890 (6)	0.0201 (9)	
C10	0.1696 (2)	0.5709 (3)	0.3354 (6)	0.0188 (9)	

### Atomic displacement parameters (Å^2^)

**Table:**

	*U*^11^	*U*^22^	*U*^33^	*U*^12^	*U*^13^	*U*^23^
Hg1	0.01515 (8)	0.01369 (8)	0.01971 (10)	−0.00045 (5)	−0.00039 (6)	−0.00147 (6)
S1	0.0154 (5)	0.0132 (4)	0.0188 (5)	0.0017 (3)	0.0049 (4)	−0.0005 (4)
S2	0.0120 (5)	0.0188 (5)	0.0271 (6)	−0.0016 (4)	0.0043 (4)	0.0004 (4)
S3	0.0367 (6)	0.0183 (5)	0.0249 (6)	−0.0089 (4)	−0.0108 (5)	0.0058 (5)
S4	0.0294 (6)	0.0140 (5)	0.0254 (6)	−0.0040 (4)	−0.0044 (5)	0.0029 (4)
N1	0.0118 (16)	0.0143 (15)	0.0133 (17)	−0.0004 (12)	0.0023 (12)	−0.0001 (13)
N2	0.0144 (16)	0.0150 (16)	0.0147 (18)	−0.0021 (12)	0.0029 (13)	0.0006 (13)
N3	0.036 (2)	0.025 (2)	0.031 (2)	−0.0093 (17)	−0.0018 (18)	−0.0027 (18)
N4	0.028 (2)	0.0228 (19)	0.027 (2)	−0.0006 (16)	−0.0031 (16)	−0.0014 (17)
C1	0.0134 (19)	0.018 (2)	0.035 (3)	0.0010 (15)	0.0055 (18)	0.0017 (19)
C2	0.0156 (19)	0.0140 (18)	0.014 (2)	0.0022 (14)	0.0011 (15)	−0.0001 (16)
C3	0.0145 (19)	0.0154 (19)	0.015 (2)	−0.0006 (14)	0.0037 (15)	−0.0014 (16)
C4	0.0134 (18)	0.0146 (18)	0.0099 (19)	0.0005 (14)	0.0013 (14)	0.0004 (15)
C5	0.0137 (18)	0.0140 (18)	0.0092 (19)	0.0008 (14)	0.0010 (14)	0.0022 (15)
C6	0.0129 (18)	0.0169 (19)	0.020 (2)	0.0005 (14)	0.0050 (15)	−0.0002 (17)
C7	0.0174 (19)	0.0133 (18)	0.017 (2)	0.0022 (15)	0.0037 (15)	0.0009 (16)
C8	0.018 (2)	0.018 (2)	0.027 (2)	0.0052 (16)	0.0064 (17)	−0.0023 (18)
C9	0.017 (2)	0.021 (2)	0.022 (2)	0.0007 (16)	0.0020 (16)	−0.0014 (18)
C10	0.0113 (19)	0.019 (2)	0.026 (2)	0.0012 (15)	0.0019 (16)	0.0042 (18)

### Geometric parameters (Å, °)

**Table:**

Hg1—S3	2.413 (1)	N4—C10	1.146 (6)
Hg1—S4	2.421 (1)	C1—C2	1.490 (5)
Hg1—N1	2.430 (3)	C1—H1A	0.9800
Hg1—N2	2.476 (3)	C1—H1B	0.9800
S1—C2	1.721 (4)	C1—H1C	0.9800
S1—C3	1.716 (4)	C3—C4	1.374 (5)
S2—C6	1.711 (4)	C3—H3	0.9500
S2—C7	1.731 (4)	C4—C5	1.455 (5)
S3—C9	1.675 (4)	C5—C6	1.356 (5)
S4—C10	1.684 (4)	C6—H6	0.9500
N1—C2	1.308 (5)	C7—C8	1.491 (5)
N1—C4	1.379 (5)	C8—H8A	0.9800
N2—C7	1.299 (5)	C8—H8B	0.9800
N2—C5	1.389 (5)	C8—H8C	0.9800
N3—C9	1.150 (5)		
			
S3—Hg1—S4	149.25 (4)	C1—C2—S1	123.0 (3)
S3—Hg1—N1	95.66 (8)	C4—C3—S1	110.0 (3)
S3—Hg1—N2	105.84 (8)	C4—C3—H3	125.0
S4—Hg1—N1	113.49 (8)	S1—C3—H3	125.0
S4—Hg1—N2	94.04 (8)	C3—C4—N1	113.7 (3)
N1—Hg1—N2	69.1 (1)	C3—C4—C5	127.4 (3)
C2—S1—C3	90.37 (19)	N1—C4—C5	118.9 (3)
C6—S2—C7	90.33 (19)	C6—C5—N2	114.4 (3)
C9—S3—Hg1	102.01 (16)	C6—C5—C4	127.7 (4)
C10—S4—Hg1	97.89 (15)	N2—C5—C4	117.9 (3)
C2—N1—C4	112.8 (3)	C5—C6—S2	110.0 (3)
C2—N1—Hg1	128.7 (3)	C5—C6—H6	125.0
C4—N1—Hg1	117.1 (2)	S2—C6—H6	125.0
C7—N2—C5	112.2 (3)	N2—C7—C8	124.8 (4)
C7—N2—Hg1	131.5 (3)	N2—C7—S2	113.0 (3)
C5—N2—Hg1	116.0 (2)	C8—C7—S2	122.2 (3)
C2—C1—H1A	109.5	C7—C8—H8A	109.5
C2—C1—H1B	109.5	C7—C8—H8B	109.5
H1A—C1—H1B	109.5	H8A—C8—H8B	109.5
C2—C1—H1C	109.5	C7—C8—H8C	109.5
H1A—C1—H1C	109.5	H8A—C8—H8C	109.5
H1B—C1—H1C	109.5	H8B—C8—H8C	109.5
N1—C2—C1	123.8 (3)	N3—C9—S3	176.2 (4)
N1—C2—S1	113.1 (3)	N4—C10—S4	177.9 (4)
			
S4—Hg1—S3—C9	−25.10 (19)	C2—S1—C3—C4	0.0 (3)
N1—Hg1—S3—C9	136.75 (17)	S1—C3—C4—N1	0.4 (4)
N2—Hg1—S3—C9	−153.45 (17)	S1—C3—C4—C5	−179.5 (3)
S3—Hg1—S4—C10	−173.66 (15)	C2—N1—C4—C3	−0.7 (5)
N1—Hg1—S4—C10	26.10 (17)	Hg1—N1—C4—C3	−168.8 (3)
N2—Hg1—S4—C10	−42.80 (16)	C2—N1—C4—C5	179.2 (3)
S3—Hg1—N1—C2	−67.8 (3)	Hg1—N1—C4—C5	11.1 (4)
S4—Hg1—N1—C2	102.2 (3)	C7—N2—C5—C6	−1.3 (5)
N2—Hg1—N1—C2	−172.6 (4)	Hg1—N2—C5—C6	−176.0 (3)
S3—Hg1—N1—C4	98.2 (3)	C7—N2—C5—C4	177.8 (3)
S4—Hg1—N1—C4	−91.8 (3)	Hg1—N2—C5—C4	3.1 (4)
N2—Hg1—N1—C4	−6.6 (3)	C3—C4—C5—C6	−10.6 (7)
S3—Hg1—N2—C7	98.2 (4)	N1—C4—C5—C6	169.5 (4)
S4—Hg1—N2—C7	−58.1 (4)	C3—C4—C5—N2	170.4 (4)
N1—Hg1—N2—C7	−171.8 (4)	N1—C4—C5—N2	−9.5 (5)
S3—Hg1—N2—C5	−88.4 (3)	N2—C5—C6—S2	0.9 (4)
S4—Hg1—N2—C5	115.3 (3)	C4—C5—C6—S2	−178.1 (3)
N1—Hg1—N2—C5	1.7 (2)	C7—S2—C6—C5	−0.3 (3)
C4—N1—C2—C1	−178.9 (4)	C5—N2—C7—C8	−179.5 (4)
Hg1—N1—C2—C1	−12.4 (6)	Hg1—N2—C7—C8	−5.8 (6)
C4—N1—C2—S1	0.6 (4)	C5—N2—C7—S2	1.0 (4)
Hg1—N1—C2—S1	167.11 (18)	Hg1—N2—C7—S2	174.69 (19)
C3—S1—C2—N1	−0.3 (3)	C6—S2—C7—N2	−0.4 (3)
C3—S1—C2—C1	179.2 (4)	C6—S2—C7—C8	−179.9 (4)
